# A qualitative exploration of attitudes towards alcohol, and the role of parents and peers of two alcohol-attitude-based segments of the adolescent population

**DOI:** 10.1186/1747-597X-9-20

**Published:** 2014-05-24

**Authors:** Meriam M Janssen, Jolanda JP Mathijssen, Marja JH van Bon-Martens, Hans AM van Oers, Henk FL Garretsen

**Affiliations:** 1 Tranzo Department, Scientific Center for Care and Welfare, Tilburg University, PO Box 90153, 5000 LE Tilburg, The Netherlands; 2 Regional Public Health Service “Hart voor Brabant”, PO Box 3166, 5203 DD ‘s-Hertogenbosch, The Netherlands; 3 The Trimbos Institute, Netherlands Institute of Mental Health and Addiction, PO Box 725, 3500 AS Utrecht, The Netherlands; 4 National Institute for Public Health and the Environment, PO Box 1, 3720 BA Bilthoven, The Netherlands

**Keywords:** Social marketing, Audience segmentation, Adolescents, Alcohol, Attitudes, Peers, Parents

## Abstract

**Background:**

An earlier study using social marketing and audience segmentation distinguished five segments of Dutch adolescents aged 12–18 years based on their attitudes towards alcohol. The present, qualitative study focuses on two of these five segments (*‘ordinaries’ and ‘ordinary sobers’*) and explores the attitudes of these two segments towards alcohol, and the role of parents and peers in their alcohol use in more detail.

**Methods:**

This qualitative study was conducted in the province of North-Brabant, the Netherlands. With a 28-item questionnaire, segments of adolescents were identified. From the *ordinaries* and *ordinary sobers* who were willing to participate in a focus group, 55 adolescents (30 *ordinaries* and 25 *ordinary sobers*) were selected and invited to participate. Finally, six focus groups were conducted with 12–17 year olds, i.e., three interviews with 17 *ordinaries* and three interviews with 20 *ordinary sobers* at three different high schools.

**Results:**

The *ordinaries* thought that drinking alcohol was fun and relaxing. Curiosity was an important factor in starting to drink alcohol. Peer pressure played a role, e.g., it was difficult not to drink when peers were drinking. Most parents advised their child to drink a small amount only. The attitude of *ordinary sobers* towards alcohol was that drinking alcohol was stupid; moreover, they did not feel the need to drink. Most parents set strict rules and prohibited the use of alcohol before the age of 16.

**Conclusions:**

Qualitative insight into the attitudes towards alcohol and the role played by parents and peers, revealed differences between *ordinaries* and *ordinary sobers*. Based on these differences and on health education theories, starting points for the development of interventions, for both parents and adolescents, are formulated. Important starting points for interventions targeting *ordinaries* are reducing perceived peer pressure and learning to make one’s own choices. For the *ordinary sobers*, an important starting point includes enabling them to express to others that they do not feel the need to drink alcohol. Starting points for parents include setting strict rules, restricting alcohol availability at home and monitoring their child’s alcohol use.

## Background

Alcohol use among European adolescents is widespread. In 2009–2010, 4% percent of 11 year old European adolescents and 8% of 13 year olds drank at least once a week [1]. Of the students aged 15–16 years, 87% have consumed alcohol and 57% drank alcohol in the last month. One fifth of all 15 year olds drank at least once a week, with over one-third (39%) of all 15–16 year olds drinking five or more drinks on one occasion (binge drinking) in the past 30 days [1,2].

There is much evidence that adolescent drinking behaviour is influenced by their parents [3]. For example, parental disapproval to the drunkenness of their child can decrease adolescents’ alcohol use [4]. On the other hand, mild parental attitudes towards adolescent drinking have been shown to result in more excessive drinking in adolescents [5]. Parents have an active role in monitoring the use of alcohol of their child [6-9], in reducing the availability of alcohol at home [7,10], and in prohibiting their child to drink alcohol [9-12]. A more stringent parental approach is likely to reduce adolescent drinking, whereas a more tolerant approach increases adolescent drinking [6,10].

Adolescents’ drinking behaviour is strongly influenced by peers [3]. On one hand, the use of alcohol by peers [4,12,13] and getting respect from peers when drinking [4] contributes to adolescent alcohol use. On the other hand, greater peer disapproval of heavy drinking results in less alcohol use and less heavy episodic drinking among adolescents [6]. Moreover, adolescents have an ambivalent view on drinking peers, i.e., they see drinking peers as relatively well adjusted but also as rebellious [14].

Due to the widespread use of alcohol by adolescents, and because of the influence of parents and peers on adolescent alcohol drinking, it is important to identify strategies which effectively target these groups to reduce alcohol-related harm to adolescents. However, until now, there has not been much evidence that alcohol education is effective in the long term [15]. A possible explanation is that adolescents are targeted as a homogeneous group in a one-size-fits-all approach in alcohol education. Therefore, we are ultimately interested in developing social marketing based interventions targeting adolescent drinking, because an important principle of social marketing is audience segmentation. This involves dividing a population into smaller and more homogeneous segments [16] based on socio-demographic data or on attitudes and behaviour [16-18]. Applying segmentation based on attitudes/behaviour enables a health educator to tailor a health education intervention to the attitudes and behaviour of a specific segment. Such a tailored health education intervention would be more appealing for this specific segment than applying a one-size-fits-all intervention [16-18]. Moreover, a specific segment would show more similarities with respect to how they might react to such tailored education efforts than the total population [18,19].

To be able to develop tailored social marketing alcohol interventions for adolescents, adolescents need to be segmented based on their attitudes/behaviour towards alcohol. Therefore, in an earlier study, after applying audience segmentation on alcohol attitudes of Dutch adolescents aged 12–18 years, we distinguished five segments: ordinaries (42%), high spirits (22%), consciously sobers (17%), ordinary sobers (11%), and socials (8%). Each segment had its own specific set of attitudes towards alcohol, based on five differentiating attitude factors: ‘aversion to intoxication’, ‘alcohol is the norm’, ‘need for approval’, ‘hedonistic associations with alcohol’, and ‘lack of interest in alcohol’ [20]. The ordinaries think alcohol is the norm, have hedonistic associations with alcohol, and have no aversion to intoxication. The high spirits are interested in alcohol, have strong hedonistic associations with alcohol, and have no aversion to intoxication. The consciously sobers do not have hedonistic associations with alcohol, are not interested in alcohol, and have an aversion to intoxication. Ordinary sobers think alcohol is the norm, have hedonistic associations with alcohol, but have an aversion to intoxication and are not interested in alcohol. Finally, the socials have a strong need for approval, do not think alcohol is the norm, are interested in alcohol, but have an aversion to intoxication [20].

With this insight, during an expert meeting with public health professionals, a well-considered choice was made to develop tailored alcohol prevention interventions for two of the five segments. This choice was based on theoretical and practical health gains. First, the ordinaries were chosen as they were the largest segment (42%) and most of them already drank alcohol; they were considered to be receptive to (tailored) interventions because they are more likely to be in control of their drinking. Second, the ordinary sobers were chosen as most of them do not (yet) drink alcohol. However, because they think alcohol is ‘normal’ and have hedonistic associations with alcohol, they are at risk of starting drinking while growing older. Therefore, the ordinary sobers were chosen with the aim to encourage continuation of their healthy non-drinking behaviour and delay the initiation of regular drinking. Because the high spirits are a high risk group that like to drink alcohol and do not set limits on the amount of alcohol consumed, it could be expected that they were also chosen for intervention development as well. However, since the high spirits were considered not to be a target group aimed at preventing to start drinking but rather a target group for treatment they were not chosen.

We already had insight into the attitudes of the ordinaries and ordinary sobers based on the quantitative audience segmentation study [20]. However, to develop tailored interventions for these two segments, in-depth insight into their alcohol attitudes and drinking behaviour, as well as the influence of parents and peers on their drinking behaviour, was needed. Alcohol attitudes and drinking behaviour may vary for different groups of adolescents; because these differences may not be revealed with quantitative data alone, qualitative data are required [21].

Therefore, for the ordinaries and ordinary sobers, the present study explores in-depth: 1) their attitudes towards alcohol, 2) their use of alcohol, 3) the role of their parents, and 4) the role of peers on their use of alcohol. The added value of the present study consists of two elements. First, the present study explored these attitudes in-depth (qualitatively) for the ordinaries and ordinary sobers. Second, the study also explored the role of parents and peers, which was not studied in the quantitative audience segmentation study. Both are expected to reveal new and important information for intervention development.

## Methods

For this qualitative study, focus groups were held among students of three Dutch high schools. This study is in compliance with the Helsinki Declaration.

### Selection of participants

Participants (12–17 years) were selected from three high schools in the working area of the Regional Public Health Service ‘Hart voor Brabant’. In this working area, all 51 high schools received an email with a brief introduction to the study and an invitation to participate. Two schools were immediately interested and another school became interested after additional details about the study and amount of time involved were provided. Of these three schools, the first is a high school offering pre-university education (students’ age 12–18 years), the second offers lower and higher general secondary education (students’ age 12–17 years), and the third offers lower general secondary education (students’ age 12–16 years).

For this study, ordinaries and ordinary sobers were required to participate in the focus groups. Therefore, the questionnaire of the audience segmentation study (consisting of 28 questions, based on the five attitude factors described earlier), with which adolescents’ segment can be determined, was used to divide adolescents into one of the five segments described above [20]. The 28 questions needed to determine students’ segment are included in Additional file 1. First, students filled in socio-demographic data (name, address, educational level, gender, and age). This information was only used by the researchers to select participants for the focus groups based on these variables and to send an invitation-letter for the focus groups to their home address. Second, students completed the 28 questions needed to determine their segment. Last, students answered a question about whether they were willing to participate in a focus group. Students filled in this questionnaire online, in class, independently from each other. Completion of this questionnaire took approximately 25 minutes.

Students of 16 classes of the first school, 11 classes of the second and 13 classes of the third school completed this questionnaire. In total, 871 students completed the questionnaire. Using SPSS, all ordinaries (n = 414) and ordinary sobers (n = 58) were identified. Then, per school, we identified all those who also wanted to participate in a focus group, resulting in 188 ordinaries and 28 ordinary sobers. Of these ordinaries and ordinary sobers, a selection per school was made by the researchers to achieve an adequate representation of age and gender. We aimed to invite 10 adolescents per focus group, in order to be able to conduct the focus groups with a minimum of five adolescents (with some drop-outs per focus group in mind). For the ordinary sobers, this was impossible because we only found 28 ordinary sobers that were willing to participate in a focus group: 13 at the first school, six at the second school, and nine at the third school. Subsequently, 30 selected ordinaries and 25 ordinary sobers were invited to participate in the focus groups.

Parents of invited ordinaries/ordinary sobers received a postal letter explaining the study and informing them of the selection of their son/daughter for participation. If parents did not agree they could mail/telephone to cancel the participation of their son/daughter. Invited ordinaries and ordinary sobers also received a postal letter with information about the date, time and location of the focus groups, and a short introduction about the focus group. They also received an email reminder two days before the focus group.

Focus group participants received a €10 cinema voucher for their participation.

### Focus groups

Six focus groups were conducted in February and March 2012, one focus group with ordinaries and one with ordinary sobers per school. Each focus group consisted of five to eight participants. A moderator and an assistant moderator (one researcher (the first author) and one assistant researcher) conducted the focus groups following a semi-structured interview guideline. The focus group explored attitudes towards alcohol, use of alcohol, the role of parents and peers on alcohol use, advised norms of alcohol use for adolescents (no alcohol consumption until reaching the minimum legal drinking age; after reaching the minimum legal drinking age, advised norms (for Dutch adolescents) are 1 glass per occasion for girls and 2 glasses per occasion for boys), activities in spare time, and alcohol prevention interventions. The focus groups lasted 70–90 min and took place at school, during school time.

Results of the first three topics (attitudes towards and use of alcohol of the ordinaries and ordinary sobers, and parental and peer influence on alcohol use of ordinaries and ordinary sobers) are presented in this manuscript. Results of the other topics are used for the development of the interventions.

### Analysis

All focus groups were audiotaped and transcribed verbatim. Analysis was done in three phases after completion of the six focus groups. All authors contributed to the analysis. First, two focus groups (one with ordinaries, one with ordinary sobers) were coded in an open way by three researchers independently from each other. In regular discussions, consensus was reached about the codes, which emerged from descriptive to analytical codes, resulting in a code list. In addition, it was discussed whether a certain quotation would be given the same code. Second, two researchers, independently from each other, coded two focus groups (one with ordinaries, one with ordinary sobers) with the code list developed in the first phase. In regular discussions, the code list was strengthened and finalised. Third, one researcher coded the last two focus groups using this final code list. The focus group coding was done manually, using Atlas Ti 7.

For this study, five Atlas Ti-families (categories of several codes) were created (see Table 1) and analysed.

**Table 1:** Overview of analysed Atlas Ti family names and codes (with operationalisation) belonging to these family names

**Atlas Ti family names**	**Codes (with operationalisation) belonging to family name**
Alcohol use of adolescent himself	- Quantity: the amount of glasses per occasion
	- Do not drink: adolescent does not drink alcohol at all
	- Only taste: adolescent ever tasted a slug/glass of alcohol
	- (More) regular: adolescent drinks alcohol on a (more) regular basis
	- Occasionally: adolescent drinks alcohol at special occasions, like birthday party, carnival, New Year.
	- Start of drinking: age at which adolescent started to drink alcohol
Attitude of adolescent himself	- Attitude of adolescent himself: attitude of adolescent towards (drinking) alcohol
Self-efficacy	- Self-efficacy: capability of adolescent of saying no to alcohol
	- Drawing limits/keeping control: drinking water/soft drink when adolescent starts to feel tipsy while drinking alcohol
	- Surroundings: influence of peers, brothers/sisters and others in the surroundings of adolescent
	- Tenability: adolescent is capable to say no to alcohol and to make his own choice
Role of parents	- Communication with parents about alcohol: conversation between parents and adolescents about (the use of) alcohol
	- Negative attitude of parents towards alcohol: parents express a negative attitude towards alcohol
	- Neutral attitude of parents towards alcohol: parents express a neutral attitude towards alcohol
	- Positive attitude of parents towards alcohol: parents express a positive attitude towards alcohol
	- Familiarity of parents with use of alcohol of child: parents know that their child drinks alcohol
	- Relationship with parents: parent-child relationship
	- Role modelling of parents: alcohol drinking behaviour of parents and role modelling of parents about alcohol
Role of peers	- Experience of alcohol use by peers: experiences of alcohol use of peers of adolescent
	- Use of alcohol by peers: peers of adolescent drink alcohol
	- Communication with peers about alcohol: conversation between adolescent and peers about (the use of) alcohol
	- Attitude of peers towards alcohol: attitude of peers of adolescent towards alcohol
	- Social influence of peers: the way the adolescent is influenced by the attitude towards or use of alcohol of peers

In the Results section, quotations are used for illustrative purposes. Each focus group (and related quotations) is identified by a unique focus group code. Codes are constructed using the letter(s) of the segment (‘O’ for ordinaries and ‘OS’ for ordinary sobers) and date of the focus group (d(d)-m-yy), e.g., O1312 indicates focus group with ordinaries on March 1, 2012 and OS15312 indicates focus group with ordinary sobers on March 15, 2012. Quotations in square brackets below indicate spoken text of the moderator.

## Results

Of the 55 selected adolescents aged 12–17 years, 37 (response rate 67%) participated in the focus groups: 17 (out of 30 invited) ordinaries and 20 (out of 25 invited) ordinary sobers. A description of age, gender and educational level of focus group participants is added in Table 2. Beforehand, parents of two adolescents and four adolescents themselves withdrew from participation. On the day of the focus group 12 participants (nine ordinaries, three ordinary sobers) failed to attend, without notification of cancelation.

**Table 2:** Socio-demographic description of focus group participants

	**Ordinaries (n = 17)**	**Ordinary sobers (n = 20)**
**Mean age**	14,3 years	13,7 years
**Number of boys**	n = 8 (47%)	n = 4 (20%)
**Educational level:**		
- High school offering pre-university education:	n = 6 (35%)	n = 8 (40%)
- Lower and higher general secondary education:	n = 6 (35%)	n = 5 (25%)
- Lower general secondary education:	n = 5 (29%)	n = 7 (35%)

To interpret the results of the focus groups in relation to the drinking age of the adolescents, it should be noted that, at the time of the study, the legal age to purchase alcohol in the Netherlands was 16 years for soft alcoholic drinks (≤15% alcohol) and 18 years for strong alcoholic drinks (≥15% alcohol).

### Attitudes towards alcohol

Ordinaries liked drinking alcohol and associated it with fun and togetherness. They liked to get tipsy, but not drunk. Ordinaries were curious about alcohol and this often led them to drink, despite their parents’ warnings not to drink alcohol before the age of 16 years.

My parents always warned me not to drink before the age of 16 because alcohol is bad for you, but I was very curious about it … just that curiosity, that rules (O28212).

I don’t want to get drunk - and when I start to feel tipsy I stop drinking alcohol and start drinking a soda or water, or something (O29212).

Ordinary sobers had a reserved attitude towards alcohol for this moment: drinking alcohol is stupid, it can ruin your life, and drinking too much can make you do things you will regret later on. Moreover, they did not like the taste of alcohol. Some ordinary sobers had a more positive attitude for the future (when reaching the minimal legal drinking age): they imagined drinking alcohol as being pleasant and that it would be nice to reach the age at which you can legally buy alcohol.

My brothers always tell stories about people who do stupid things that they’ll regret. You also hear stories - and also, if you drink a lot of alcohol or drink yourself into a coma - you get talked about and you don’t want that … (OS15312).

My grandfather always likes to drink a glass of alcohol while I drink a glass of iced tea, which I think has a better taste - and is also better (for my health). (OS15312).

### Use of alcohol

Most of the ordinaries drank alcohol and, in their opinion, they did not drink much. For them, ‘not much’ was two—eight glasses on one occasion. Being tipsy was their limit, then they switched from alcohol to water/soft drink. Ordinaries drank their first glass of alcohol when aged 14–15 years.

I don’t drink a little - but also, not a lot. When I go out, four glasses of beer – maybe up to six. If we’re really having a good time, we also drink shots - quite a lot (O28212).

I drink, I think, six to eight glasses of beer in one evening (O29212).

Most of the ordinary sobers did not drink alcohol and did not feel the need to drink. Some had tasted alcohol at some time and some drank alcohol once in a while. Ordinary sobers did not really like the taste of alcohol. The ordinary sobers who drank, drank one—three glasses on one occasion.

Yes, I’m allowed to drink, - well, I don’t feel the need to drink alcohol (OS15312).

Once, at New Year, my mother or my father gave me a glass of champagne - but I really didn’t like the taste (OS15312).

### Role of parents

Most ordinaries reported that they did not talk with their parents about (the use of) alcohol. Some ordinaries told their parents that they drank alcohol, whereas some did not (always) tell their parents. According to the ordinaries, the mothers of some ordinaries did not agree with the drinking of their child, while the fathers seemed less concerned. If ordinaries and parents talked about alcohol, the conversations were mainly about school performance, and the amount of alcoholic drinks and the kind of alcoholic drinks (strong drinks/liquor). Some ordinaries reported that their parents set rules, like forbidding their son/daughter to drink alcohol before age 16 years. Some ordinaries reported that their parents did not set rules; ordinaries reported that their parents only advised them not to drink too much, or to only start drinking after reaching the legal age of 16 years.

My mother actually doesn’t know and my father … he used to drink, himself. So, when I don’t drink too much, he doesn’t care (O1312).

No, most of the time, when I go to a party, they just say, yes, don’t drink too much, and I stick to that, or … I know for myself.

[Yes, so she advises you, but you can decide for yourself?]

Yes (O1312).

My mother still thinks that I don’t drink a lot - when I’ve been out she asked me how many drinks I had - and I told her two beers - or something. She got into a panic, but, actually, I drank eight beers - or something like that. But I don’t tell her because she’ll go out of her mind, I think; it really bothers my mother. And yes, actually it’s weird, because my brother went out every week when he was 16 and it didn’t bother her at all. (O29212).

Ordinary sobers reported that they and their parents seldom talked about (the use of) alcohol. First, ordinary sobers reported that drinking alcohol was not an issue for them, and their parents knew this and trusted their children. Second, ordinary sobers reported that most of their parents prohibited the use of alcohol and, therefore, they were not allowed to drink until age 16. Ordinary sobers respected these parental rules and, therefore, did not drink alcohol. However, some ordinary sobers told that they were allowed to drink a small glass on special occasions, e.g., carnival time, or a birthday at home.

It doesn’t cross my mind to start drinking alcohol - and my parents know this, so they don’t start talking about alcohol. But … they just know whatever I know, that I will never drink alcohol before the age of 16, so … they don’t talk to me about alcohol (OS15312).

[Did you make agreements with your parents?]

No, they just know that I won’t drink alcohol.

[No? You also told us that they do not drink alcohol themselves]

No…at home, no alcohol is available.

[So, you did not make concrete agreements?]

No … yes - they think drinking alcohol is not wise, and neither do I (OS22312).

### Role of peers

For the ordinaries, peer pressure played an important role. They considered it difficult to say ‘no’ to alcohol when peers drank alcohol; with drinking peers, ordinaries drank alcohol more often than with their non-drinking peers. However, some ordinaries also indicated that, on occasion, they would drink a soft drink while their peers drank alcohol. When peers drank soda, then ordinaries also drank soda; they did not drink alcohol when being the only one. Ordinaries stated that alcohol was not a necessary ingredient for having fun. According to them, it should be emphasised that someone does not have to be ashamed of drinking water or a soft drink.

The ordinaries saw their drinking peers as happy, more relaxed and noisier than non-drinking peers. Moreover, it was stated that adolescents aged ≤16 years (i.e., under the legal drinking age and not allowed to buy alcohol) asked adolescents aged ≥16 years to buy alcohol for them, thereby by-passing the rules. Generally, ordinaries did not talk about (drinking) alcohol.

I think a little,…when everybody is - I would not drink a lot but would think, well, then I’ll also drink a glass of alcohol; it’s easier than when they don’t drink.

[All right, so if everybody drinks a soft drink, it makes it easier for you to drink a non-alcoholic drink. But when your peers are drinking, you would be inclined to …]

Yes, I’d be inclined to drink alcohol (O1312).

[You say, when everybody is having a beer, it’s difficult for you to have a cola…]

*Yes, I stuck to a soft drink for a long time, in grade 8 (13*–*14 year olds) everybody already drank a lot of alcohol. My friends as well … I can’t remember my turning point, but suddenly I thought … one alcoholic drink is okay (O29212).*

Most of the ordinary sobers turned down an alcoholic drink when it was offered. Friends respected their choice not to drink alcohol. However, one ordinary sober reported she felt insecure when alcohol was offered which she did not want, whereas she also felt happy because she managed to say ‘no’ to drinking this alcohol. Two ordinary sobers accepted an offered alcoholic drink; for them it was too difficult to say ‘no’. It frightened ordinary sobers when their friends under the age of 16 drank alcohol, and they felt uncomfortable with drunken friends whilst they were sober. According to the ordinary sobers, self-confidence was an important factor for refusing alcohol when it was offered.

I generally feel uncomfortable when friends of mine, like at carnival time, get offered an alcoholic drink … and yes, I was the only one that turned it down. So, I felt insecure … but also happy that I turned it down (OS15312).

Last school party, all of a sudden my friends were drinking alcohol … and that shocked me (OS15312).

Yes and no: yes, one of my best friends - she drinks alcohol and that makes me … join her - when I happen to be at her place (OS22312).

## Discussion

This study showed new insights into the differences in alcohol-attitudes and alcohol drinking behaviour between the ordinaries and ordinary sobers. Ordinaries had a positive attitude towards alcohol, associated it with fun and started drinking because they were curious. Most of the ordinaries already drank alcohol. Most ordinary sobers did not drink alcohol, did not like the taste of alcohol, nor did they feel the need to drink. They had a reserved attitude towards alcohol. Moreover, this study showed that parents played a different role in alcohol education for these two segments. Although parents of some ordinaries set rules about alcohol use, the majority only advised their son/daughter not to drink too much or to start drinking only after reaching the minimum legal drinking age. Ordinary sobers reported that their parents generally set rules about not drinking alcohol until age 16, which were respected by the ordinary sobers. Last, it was found in this study that peers also influenced the attitudes and alcohol use of the ordinaries and ordinary sobers in a different way. Ordinaries experienced peer pressure and were inclined to drink alcohol when peers were drinking, whereas most ordinary sobers were able to resist an offered alcoholic drink, a choice that was respected by peers.

For the ordinary sobers, there appeared to be a difference in their attitude towards alcohol in the results found in this current qualitative study and in the earlier quantitative audience segmentation study. In the focus groups, the ordinary sobers expressed a reserved attitude towards alcohol and they were not interested in alcohol. However, results from the earlier quantitative audience segmentation study [20] showed that the ordinary sobers appeared to have a positive attitude towards alcohol: thinking about alcohol made them think of the having fun, of letting go, and of adulthood. A possible explanation for these differences in attitudes is that the positive attitude found in the audience segmentation study is a future-directed attitude, whereas the attitude explored in the focus groups described the attitude of the ordinary sobers for this moment, influenced by the strict rules of parents and the fact that ordinary sobers are not allowed to drink alcohol before the age of 16. Because of this, their attitude does not lead to intentions to drink and to actual alcohol drinking behaviour.

Other qualitative studies have found alcohol-related attitudes of adolescents that are in agreement with that found in the ordinaries segment. Adolescents appeared to drink alcohol to relax, to have fun, and to belong to the group [22] and alcohol was seen by adolescents as a central marker of maturity and was used to gain social recognition [23]. According to a review of drinking motives, most young people drink because of social motives being either positive (social camaraderie) or negative (peer pressure, not to feel left out) [24]. If we translate the results of these studies to the present study, it appears that the ordinaries drink because they experienced peer pressure, which can be seen as a struggle for social recognition and a need to belong to the group. The attitudes of the ordinary sobers were not reflected in the review of drinking motives [24], because only drinking adolescents were studied in this review study. The added value of the current study was that we found differences in alcohol-attitudes between the ordinaries and the ordinary sobers by applying audience segmentation. These differences will enable us to tailor social marketing alcohol health education to these different attitudes of the two segments.

Many of the constructs emerging from the focus groups are aligned with key theories that explain lifestyle behaviours, like drinking alcohol. Key theories for explaining lifestyle behaviours are the “Theory of Planned Behavior” [25,26], “Drinking Refusal Self-Efficacy” [27-29] and the “Social Cognitive Theory” [30]. These theories are also useful to underpin intervention development. The “Theory of Planned Behavior” states that behavioural intentions are influenced by three determinants: the attitude towards the behaviour, the subjective norm, and the perceived behavioural control. Behaviour (change) is influenced by the intentions [25,26]. The second theoretical construct to explain the results of this study is the “Drinking Refusal Self-Efficacy”-theory [27-29]. Drinking refusal self-efficacy is a person’s belief about his/her ability to refuse alcohol in certain situations [29] and, according to this theory, drinking refusal self-efficacy is a predictor of alcohol consumption [27]. A third and final theoretical construct to explain the results of this study is the “Social Cognitive Theory” [30], which states that (expectations of) ‘environmental events’, ‘personal factors’, and ‘behaviour’ influence each other continually.

Based on the results of the present study and the theoretical constructs, starting points for preventive alcohol interventions for adolescents can be formulated, based on the specific insights into the ordinaries and ordinary sobers. It is important that interventions for adolescents are aimed at the ordinaries and ordinary sobers, as well as their peers, as peers might help them to say ‘no’ to alcohol or to respect that the ordinary sobers do not feel the need to drink alcohol.

Starting points for the ordinaries are based on the subjective norm and on the perceived behavioural control. Ordinaries experience social pressure of drinking peers, and their perceived behavioural control of drinking a soft drink while peers drink alcohol is low. Therefore, an important starting point for an intervention for the ordinaries is peer pressure. Another important starting point is increasing their perceived behavioural control; this can be done by emphasizing that it is important to make your own choice and by practicing this in skills-training. Besides, this can be done by creating respect for each other’s choices, even when ordinaries make their own choices which might differ from those of their peers. Some ordinaries were drinking large volumes of alcohol on one occasion. Drinking (a lot of) alcohol at a young age can for example harm the immature and developing brains of adolescents and can result in alcohol poisoning. Therefore, it is important to incorporate knowledge about the harm alcohol can cause in an intervention for the ordinaries.

The starting point for the ordinary sobers is based on their reserved attitude towards alcohol and is aimed at continuing this reserved attitude. The ordinary sobers do not (yet) drink alcohol. For this moment, they do not feel the need to drink alcohol. However, because they think alcohol is the norm and have hedonistic associations with alcohol [20], they might start drinking alcohol when turning older. Based on these insights, a starting point for an intervention for ordinary sobers is to enable them to express to their peers that they do not feel the need to drink alcohol and to create respect for their choice not to drink alcohol.

It is advised to also focus on the parents in interventions. This study showed that parents of both groups conducted their alcohol education role in different ways. Parents of the ordinaries rarely set strict rules about alcohol and advised their children not to drink too much, whereas parents of the ordinary sobers set clear rules about not drinking until reaching age 16 years.

In general, parents do their best to minimise harm and promote healthy alcohol behaviour in their children [31,32]. Alcohol use by ordinaries and ordinary sobers can be influenced by parental alcohol education. Parental measures (e.g., monitoring their child’s use of alcohol, restricting the availability of alcohol at home, and setting rules about the use of alcohol) are important and effective measures [6-12]. Because parents can influence the attitude and alcohol use of their child by applying such measures, these should be the starting points for interventions for parents of both ordinaries and ordinary sobers. For parents of ordinaries, it could be stressed that setting strict rules and maintaining these rules might help their son/daughter not to drink (too much) alcohol. For the parents of the ordinary sobers, it could be stressed that they continue setting rules after their son/daughter reaches the minimum legal drinking age, i.e., rules about the amount of days/week the ordinary sober is allowed to drink alcohol or of the amount of glasses an ordinary sobers consumes per occasion.

Besides, it is important to educate the parents of both the ordinaries and the ordinary sobers about the short-term and long-term alcohol-related harm of adolescent alcohol drinking, i.e., alcohol poisoning, brain damage, the risk of conducting risky sexual behaviour, neurological damage, and having an increased risk of becoming dependent or addicted in later life.

### Study limitations

Of the 55 invited adolescents, 18 did not participate due to withdrawal by the adolescents themselves (four in advance, 12 not showing on the day of the focus group) or because their parents did not want them to participate, which was the case for two adolescents.

Focus groups took place at school during school time. Possible reasons for withdrawal or not showing up are: having an examination/test, wanting to follow lessons due to poor school results, forgetting the date (despite an email reminder), and/or being ill. Of the 18 non-participants, 13 were ordinaries and five were ordinary sobers. In total, 57% of the invited ordinaries and 80% of the invited ordinary sobers participated in the focus groups. We did not ask for reasons for withdrawal. Besides the above-mentioned reasons, a possible explanation for withdrawal for the ordinaries (based on their attitudes about alcohol) is that they might be less inclined to participate in a focus group and share their opinion/experiences about alcohol (use).

The researchers composed the focus groups with a good mix of age and gender. Therefore, boys and girls, of a younger and older age, participated together in a focus group. It might be possible that the composition of the focus groups has hindered participants to be totally honest about their opinion. However, we did not notice so; all participants participated actively in the focus groups. Besides, the researchers paid attention that all participants could and did express their own opinion. The segmentation based on alcohol attitudes might have helped in this; the participants of one focus group shared the same attitudes towards alcohol. Moreover, it might be possible that results differ per school and, thus, per educational level and were influenced by an overrepresentation of girls in the focus groups with the ordinary sobers. However, because the segments were based on attitudes towards alcohol, and not on socio-demographic variables, this seems less relevant. Finally, it might be possible that group dynamics (participants know each other or even might be friends) influenced the results. During the focus groups, some participants seemed to know each other, however, researchers did not observe the participants to be friends. Each participant answered upon his/her own individual opinion.

This type of qualitative research provides deeper insight into the attitudes of ordinaries and ordinary sobers towards alcohol, and the role of their parents and peers in the Netherlands. In order to explore cross-cultural applications of this study, more research is needed.

## Conclusions

Qualitative insight into the attitudes and use of alcohol of the ordinaries and ordinary sobers, and the role of their parents and peers has revealed new differences between these two segments. Most of the ordinaries already drink alcohol. Ordinaries experience peer pressure and are inclined to drink alcohol when peers are drinking. The majority of the parents of ordinaries only advised their son/daughter not to drink too much or to start drinking only after reaching the minimum legal drinking age.

Most ordinary sobers do not drink alcohol nor do they feel the need to drink. Ordinary sobers respected parental rules about not drinking alcohol until age 16. These differences led to different starting points for interventions. For intervention development, it is advised that an intervention is aimed at adolescents, as well as at their parents and peers. Starting points for an intervention for ordinaries are reducing peer pressure and asking peers to respect their friends’ choices which might differ from their choices. A starting point for an intervention for ordinary sobers is prompting them to confirm that they do not feel the need to drink. Starting points for an intervention for parents are monitoring the use of alcohol of their child, restricting the availability of alcohol at home, and setting clear rules about alcohol use.

## Competing interests

The authors declare that they have no competing interests.

## Authors’ contributions

MJ was responsible for data collection and reporting of the study results. All authors participated in the analysis and interpretation of the findings, reviewed the manuscript, and approved the final manuscript.

## Supplementary Material

Additional file 1 The 28 questions with which adolescents’ segments can be determined. Click here for file

## References

[B1] Currie C Zanotti C Morgan A Currie D de Looze M Roberts C Samdal O Smith ORF Barnekow V Social determinants of health and well-being among young people. Health behaviour in school-aged children (HBSC) study: international report from the 2009/2010 survey Health Policy for Children and Adolescents, No. 6 2012 Copenhagen: World Health Organization Regional Office for Europe

[B2] European Monitoring Centre for Drugs and Drug Addiction Summary 2011 ESPAD Report. Substance use Among Students in 36 European Countries 2012 Luxembourg: Publications Office of the European Union

[B3] Cleveland MJ Feinberg ME Greenberg MT Protective families in high- and low-risk environments: implications for adolescent substance use J Youth Adolesc 2010 39 2 114 126 10.1007/s10964-009-9395-y 20084558 PMC2809936

[B4] Kristjansson AL Sigfusdottir ID James JE Allegrante JP Helgason AR Perceived parental reactions and peer respect as predictors of adolescent cigarette smoking and alcohol use Addict Behav 2010 35 3 256 259 10.1016/j.addbeh.2009.10.002 19892468

[B5] Mares SHW van der Vorst H Engels R Lichtwarck-Aschoff A Parental alcohol use, alcohol-related problems, and alcohol-specific attitudes, alcohol-specific communication, and adolescent excessive alcohol use and alcohol-related problems: an indirect path model Addict Behav 2011 36 3 209 216 10.1016/j.addbeh.2010.10.013 21084165

[B6] Walls TA Fairlie AM Wood MD Parents do matter: a longitudinal two-part mixed model of early college alcohol participation and intensity J Stud Alcohol Drugs 2009 70 6 908 918 19895767 10.15288/jsad.2009.70.908

[B7] Schelleman-Offermans K Kuntsche E Knibbe RA Associations between drinking motives and changes in adolescents’ alcohol consumption: a full cross-lagged panel study Addiction 2011 106 7 1270 1278 21375645 10.1111/j.1360-0443.2011.03423.x

[B8] Barnes GM Reifman AS Farrell MP Dintcheff BA The effects of parenting on the development of adolescent alcohol misuse: a six-wave latent growth model J Marriage Fam 2000 62 175 186 10.1111/j.1741-3737.2000.00175.x

[B9] Koning IM Engels R Verdurmen JEE Vollebergh WAM Alcohol-specific socialization practices and alcohol use in Dutch early adolescents J Adolesc 2010 33 1 93 100 10.1016/j.adolescence.2009.05.003 19520421

[B10] Koning IM van den Eijnden RJ Verdurmen JE Engels RC Vollebergh WA A cluster randomized trial on the effects of a parent and student intervention on alcohol use in adolescents four years after baseline: no evidence of catching-up behavior Addict Behav 2013 38 4 2032 2039 10.1016/j.addbeh.2012.12.013 23391851

[B11] van der Vorst H Engels R Burk WJ Do parents and best friends influence the normative increase in adolescents’ alcohol use at home and outside the home? J Stud Alcohol Drugs 2010 71 1 105 114 20105420 10.15288/jsad.2010.71.105

[B12] Chan GCK Kelly AB Toumbourou JW Hemphill SA Young RM Haynes MA Catalano RF Predicting steep escalations in alcohol use over the teenage years: age-related variations in key social influences Addiction 2013 108 11 1924 1932 23834266 10.1111/add.12295 PMC3797268

[B13] Musher-Eizenman DR Holub SC Arnett M Attitude and peer influences on adolescent substance use: the moderating effect of age, sex, and substance J Drug Educ 2003 33 1 1 23 10.2190/YED0-BQA8-5RVX-95JB 12773022

[B14] Spijkerman R van den Eijnden R Vitale S Engels R Explaining adolescents’ smoking and drinking behavior: the concept of smoker and drinker prototypes in relation to variables of the theory of planned behavior Addict Behav 2004 29 8 1615 1622 10.1016/j.addbeh.2004.02.030 15451128

[B15] Alcohol and Public Policy Group Alcohol: no ordinary commodity-a summary of the second edition Addiction 2010 105 5 769 779 20331569 10.1111/j.1360-0443.2010.02945.x

[B16] Forthofer MS Bryant CA Using audience-segmentation techniques to tailor health behavior change strategies Am J Health Behav 2000 24 1 36 43 10.5993/AJHB.24.1.6

[B17] Slater MD Theory and method in health audience segmentation J Health Commun 1996 1 3 267 283 10.1080/108107396128059 10947364

[B18] Moss HB Kirby SD Donodeo F Characterizing and reaching high-risk drinkers using audience segmentation Alcohol Clin Exp Res 2009 33 8 1336 1345 10.1111/j.1530-0277.2009.00963.x 19413650 PMC3016848

[B19] Boslaugh SE Kreuter MW Nicholson RA Naleid K Comparing demographic, health status and psychosocial strategies of audience segmentation to promote physical activity Health Educ Res 2005 20 4 430 438 10.1093/her/cyg138 15572439

[B20] Mathijssen JJP Janssen MM Van Bon-Martens MJH Van de Goor LAM Adolescents and alcohol: an explorative audience segmentation analysis BMC Public Health 2012 12 742 doi: 10.1186/1471-2458-12-742 10.1186/1471-2458-12-742 PMC3490719 22950946

[B21] Strunin L Assessing alcohol consumption: developments from qualitative research methods Soc Sci Med 2001 53 215 226 10.1016/S0277-9536(00)00332-4 11414388

[B22] Samardžić S Bujšić G Kožul K Tadijan D Drinking in adolescents - qualitative analysis Coll Antropol 2011 35 1 123 126 21661361

[B23] Demant J Järvinen M Constructing maturity through alcohol experience - focus group interviews with teenagers Addiction Res Theor 2006 14 6 589 602 10.1080/16066350600691683

[B24] Kuntsche E Knibbe RA Gmel G Engels R Why do young people drink? A review of drinking motives Clin Psychol Rev 2005 25 841 861 10.1016/j.cpr.2005.06.002 16095785

[B25] Ajzen I Kuhl J, Beckmann J From intentions to actions: a theory of planned behavior Action Control: From Cognition to Behavior 1985 Heidelberg, Berlin: Springer-Verlag 11 39

[B26] Ajzen I The theory of planned behavior Organ Behav Hum Decis Process 1991 50 179 211 10.1016/0749-5978(91)90020-T

[B27] Oei TPS Burrow T Alcohol expectancy and drinking refusal self-efficacy: a test of specificity theory Addict Behav 2000 25 4 499 507 10.1016/S0306-4603(99)00044-1 10972442

[B28] Oei TPS Lee Jarmin C Alcohol expectancies, drinking refusal self-efficacy and drinking behaviour in Asian and Australian students Drug Alcohol Depend 2007 87 281 287 10.1016/j.drugalcdep.2006.08.019 16996231

[B29] Oei TPS Morawska A A cognitive model of binge drinking: the influence of alcohol expectancies and drinking refusal self-efficacy Addict Behav 2004 29 159 179 10.1016/S0306-4603(03)00076-5 14667427

[B30] Bandura A Social Foundations of Thought and Action: A Social Cognitive Theory 1986 New Jersey: Englewood Cliffs

[B31] Bourdeau B Miller B Vanya M Duke M Ames G Defining alcohol-specific rules among parents of older adolescents: moving beyond no tolerance J Fam Commun 2012 12 2 111 128 10.1080/15267431.2011.561140 23204931 PMC3509802

[B32] Gilligan C Kypri K Parent attitudes, family dynamics and adolescent drinking: qualitative study of the Australian parenting guidelines for adolescent alcohol use BMC Public Health 2012 12 491 doi:10.1186/1471-2458-12-491 10.1186/1471-2458-12-491 PMC3461436 22747699

