# Supplementary material for: A qualitative exploration of attitudes towards alcohol, and the role of parents and peers of two alcohol-attitude-based segments of the adolescent population
Source: Subst Abuse Treat Prev Policy. 2014 May 24;9:20. doi: 10.1186/1747-597X-9-20 (PMC4045867; doi:10.1186/1747-597X-9-20)
Supplement: Additional file 1 — The 28 questions with which adolescents’ segments can be determined. [file 1747-597X-9-20-S1.docx]

**Additional file 1: the 28 questions with which adolescents’ segments can be determined.**

Adolescents could answer in a 5-point Likert scale, answer categories ranging from ‘totally disagree’ to ‘totally agree’

1. The opinion of my parents***** is important to me

2. It's important to me that my friends have a good opinion of me

3. It seems only natural to me to keep to my parents’ rules

4. For me it's important not to be different from other people

5. My parents take my opinion seriously

6. I learn from my mistakes

7. My friends would be embarrassed if I got drunk

8. People are more fun when they've been drinking

9. Stronger action should be taken against alcohol misuse

10. I would be embarrassed if I got drunk myself

11. People become annoying when they've been drinking

12. I would find it amusing if one of my friends got drunk

13. I would be embarrassed if one of my friends got drunk

14. Alcohol is more for boys than for girls

15. Drinking alcohol is more fun when it's not allowed

16. I can imagine that you don't want to be seen with a soft drink when everyone else is drinking alcohol

17. My parents would be embarrassed if I got drunk

18. It’s weird if an adult never drinks alcohol

19. Since it's legal to buy alcohol once you are 16, it must be less damaging from that age

20. I think it's exciting to be drunk

21. Alcohol makes me think of having fun

22. Alcohol makes me think of adulthood

23. Alcohol makes me think of the weekend

24. Alcohol makes me think of a drink with a meal

25. Alcohol makes me think of relaxing

26. Alcohol makes me think of letting go

27. Alcohol makes me think: Don't like the taste

28. Alcohol makes me think: Not for me

*The word “parents” can also be read as parent or carer(s).
